# Soluble Epoxide Hydrolase Activity Determines the Severity of Ischemia-Reperfusion Injury in Kidney

**DOI:** 10.1371/journal.pone.0037075

**Published:** 2012-05-10

**Authors:** Jung Pyo Lee, Seung Hee Yang, Hee-Yoon Lee, Bora Kim, Joo-Youn Cho, Jin Ho Paik, Yun Jung Oh, Dong Ki Kim, Chun Soo Lim, Yon Su Kim

**Affiliations:** 1 Department of Internal Medicine, Seoul National University Boramae Medical Center, Seoul, Korea; 2 Seoul National University Kidney Research Institute, Seoul, Korea; 3 Department of Internal Medicine, Seoul National University College of Medicine, Seoul, Korea; 4 Department of Chemistry, Korea Advanced Institute of Science and Technology, Daejeon, Korea; 5 Department of Pharmacology and Clinical Pharmacology, Seoul National University College of Medicine, Seoul, Korea; 6 Department of Pathology, Seoul National University College of Medicine, Seoul, Korea; University of Florida, United States of America

## Abstract

Soluble epoxide hydrolase (sEH) in endothelial cells determines the plasma concentrations of epoxyeicosatrienoic acids (EETs), which may act as vasoactive agents to control vascular tone. We hypothesized that the regulation of sEH activity may have a therapeutic value in preventing acute kidney injury by controlling the concentration of EETs. In this study, we therefore induced ischemia-reperfusion injury (IRI) in C57BL/6 mice and controlled sEH activity by intraperitoneal administration of the sEH inhibitor 12-(3-adamantan-1-ylureido)-dodecanoic acid (AUDA). The deterioration of kidney function induced by IRI was partially moderated and prevented by AUDA treatment. In addition, AUDA treatment significantly attenuated tubular necrosis induced by IRI. Ischemic injury induced the down-regulation of sEH, and AUDA administration had no effect on the expression pattern of sEH induced by IRI. In vivo sEH activity was assessed by measuring the substrate epoxyoctadecenoic acid (EpOME) and its metabolite dihydroxyoctadec-12-enoic acid (DHOME). Ischemic injury had no effects on the plasma concentrations of EpOME and DHOME, but inhibition of sEH by AUDA significantly increased plasma EpOME and the EpOME/DHOME ratio. The protective effect of the sEH inhibitor was achieved by suppression of proinflammatory cytokines and up-regulation of regulatory cytokines. AUDA treatment prevented the intrarenal infiltration of inflammatory cells, but promoted endothelial cell migration and neovascularization. The results of this study suggest that treatment with sEH inhibitors can reduce acute kidney injury.

## Introduction

Ischemia-reperfusion injury (IRI) is the leading cause of acute kidney injury (AKI), which is associated with a high mortality [1], [2]. Although the pathogenesis of renal IRI has not been fully clarified, hypoxic cell injury both during the ischemic phase and following inflammatory responses in the reperfusion phase are known to play roles [3], [4].

Epoxyeicosatrienoic acids (EETs) are metabolites of arachidonic acid and are considered likely to represent one of the endothelium-derived hyperpolarizing factors that mediate the vascular effects of vasoactive hormones [5]–[7]. Renal EETs are involved in renal blood flow regulation and long-term arterial blood pressure control by acting as an endothelium-derived hyperpolarizing factor on preglomerular vascular smooth muscle cells to dilate the afferent arterioles [8]. Hence, renal and cardiovascular diseases are associated with decreased renal and vascular concentrations of EETs [9]. EETs also possess potent anti-inflammatory [10] and fibrinolytic [11] effects. Soluble epoxide hydrolase (sEH) catalyses the degradation of EETs to their corresponding diols, and thus plays a central role in the regulation of EET concentrations [12]. We recently demonstrated a genetic effect of sEH encoded by the *EPHX2* gene on the progression of IgA nephropathy [13] and on renal allograft survival [14], and some studies have shown a protective effect of sEH inhibitors against IRI in stroke [15], [16] and ischemia-induced myocardial damage [17]–[19].

In this study, we hypothesized that increasing the concentration of EETs by inhibiting sEH could represent a promising therapeutic target for AKI. We therefore investigated the effects of the sEH inhibitor 12-(3-adamantan-1-ylureido)-dodecanoic acid (AUDA) on the regulation of intrarenal inflammation and promotion of neovascularization in a mouse renal IRI model.

## Materials and Methods

### Experimental animals and chemicals

Male C57BL/6 mice weighing 20–22 g and 7–8 weeks old were purchased from Orient Company (Seoul, Korea). All the mice were raised in a pathogen-free animal facility. All experiments were performed under the approval of the Institutional Animal Care and Use Committee of Clinical Research Institute at Seoul National University Hospital and in accordance with the National Research Council ‘Guidelines for the Care and Use of Laboratory Animals’ [20]. The adamantyl alkyl urea-based sEH inhibitor AUDA was synthesized by one of the co-authors, as previously reported [21]. AUDA was dissolved in (2-hydroxypropyl)-β-cyclodextrin (cyclodextrin; Sigma Chemical Co., St Louis, MO, USA) at 5 mg/mL [22].

### Induction of renal IRI

An established murine renal IRI model was used [23]. Briefly, mice were anesthetized by intraperitoneal injection of ketamine (100 mg/kg body weight) and pentobarbital sodium (Nembutal, 50 mg/kg body weight; Abbott, Wiesbaden, Germany). Following an abdominal midline incision, both renal pedicles were bluntly dissected and clamped with a microvascular clamp (Roboz Surgical Instrument, Gaithersburg, MD, USA) for 30 min. During the procedure, 2 mL of sterile saline at 40°C (1 mL during ischemia and 1 mL during reperfusion) were instilled into the peritoneal cavity. After the clamps were removed, the wounds were sutured and the mice were allowed to recover, with free access to chow and water. Adequate reperfusion was confirmed with the naked eye after declamping. The mice were placed on a heating pad (40°C) throughout the procedure, and blood pressure was measured using a non-invasive blood pressure system (Kent Scientific Corp., Chicago, IL, USA). Sham-operated mice underwent identical surgical procedures, except for clamping of the renal pedicles. The sEH inhibitor, AUDA (10 mg/kg), or β-cyclodextrin (300 µL/mouse) was administered intraperitoneally 1 h before ischemia-reperfusion surgery. The dose of AUDA was selected according to previously published articles [16], [22]. Blood samples were obtained from the tail vein before, and at 24 h and 48 h after renal IRI. Mice were sacrificed 48 h after reperfusion. Renal function in mice subjected to ischemia surgery was evaluated by measuring creatinine (Cr) concentrations. Serum Cr concentrations (mg/dL) were measured using the modified Jaffé rate reaction and an autoanalyzer (Hitachi Chemical Industries Ltd, Osaka, Japan). Five to six mice were used for each group and three independent experiments were performed for each procedure.

### Tissue histology

Mice were killed at 48 h after IRI and kidneys were harvested after exsanguination. Tissue samples were fixed with 10% buffered formalin, followed by paraffin embedding. Paraffin sections (4 µm) were then stained with periodic acid-Schiff reagent. The degrees of tubular injury were scored by a renal histologist blinded to the sample groups. Cell loss and necrosis were graded according to five levels, based on the percentages of injured tubules in the cortex and the two different medullary areas (outer medulla and inner medulla) respectively (1: <10%; 2: 10–25%; 3: 25–50%; 4: 50–75%; 5: >75%). Injured tubules were scored according to the proportions of necrotic tubules and tubular casts relative to the total number of tubules [23].

### Cell culture

Human umbilical vein endothelial cells (HUVECs) (Cambrex, Walkersville, MD, USA) were cultured for four to six passages in EGM-2 MV (Clonetics Corp., San Diego, CA, USA) supplemented with 10% fetal bovine serum, 1 mg/mL hydrocortisone, 12 mg/mL bovine brain extract, 50 mg/mL gentamycin, 50 ng/mL amphotericin B, and 10 ng/mL epidermal growth factor (EGF). After 3 days of culture, cells were detached from the dishes by the addition of a 3 mM EDTA solution and a minimal amount of trypsin. Cells (2×10^5^/well) were then placed in 8-well chamber slides with serum-free medium for 24 h and washed twice with phosphate-buffered saline (PBS). Cells were incubated with or without AUDA (10 µM) under hypoxic (1% O_2_) or normoxic conditions (20% O_2_) for 24 h. The expression levels of sEH, p53, hypoxia inducible factor (HIF)-1α, vascular endothelial growth factor (VEGF) receptor-2 (KDR), and c-kit were measured by confocal microscopy, as described below.

### Confocal microscopy

Confocal microscopy was performed using an LSM 510 Meta laser confocal microscope (Carl Zeiss, Jena, Germany). For the immunofluorescence study, paraffin-embedded samples were collected and cut into 4-µm slices. The slices were deparaffinized and hydrated using xylene and ethanols, then stained with primary antibodies in a blocking reagent overnight at 4°C. The antibodies used were as follows: rabbit sEH (Santa Cruz Biotechnology, Santa Cruz, CA, USA), rat CD31 (BD Biosciences, Franklin Lakes, NJ, USA), rat F4/80 (abcam, Cambridge, UK), mouse CD3 (BD Biosciences), and rabbit myeloperoxidase (MPO) (abcam). A second layer of Alexa Fluor® 488-conjugated goat anti-rabbit antibody (Molecular Probes, Eugene, OR, USA), Alexa Fluor® 555-conjugated anti-rat antibody, and Alexa Fluor® 555-conjugated anti-mouse antibody, respectively, were used as secondary antibodies. All sections were washed and incubated for an additional 5 min with 4′,6-diamidino-2-phenylindole (DAPI) for counterstaining. Primary antibodies were omitted from the sections for the negative control.

In another set of experiments, the kidneys were snap-frozen in OCT embedding medium (Miles Inc., Elkhart, IN, USA), cooled to −80°C and cut into 5-µm-thick sections using a cryostat (Leica, Heidelberger, Germany). Frozen sections were fixed for 10 min in cold acetone. Intrarenal expression of c-kit (Abcam, Cambridge, UK) and CD31 were measured by confocal microscopy.

Immunofluorescence analysis of HUVECs in culture was carried out as follows. Briefly, cultured HUVECs in 8-well-chamber slides were fixed in 2% paraformaldehyde and stained for sEH, p53 (Cell Signaling Technology, Beverly, MA, USA) and HIF-1α (Novus Biologicals, Inc., Littleton, CO, USA). Immunofluorescence analyses of KDR and c-kit were carried out using cytospin preparations, prepared by centrifugation of 200 µL of cell suspension for 3 min at 1000 rpm in a Shandon Cytospin III cytocentrifuge onto conventional glass slides. The slides were then fixed with 2% paraformaldehyde for 5 min and washed twice with PBS. Immunostaining was performed using mouse anti-human KDR (Sigma Chemical Co.) and rabbit anti-human c-kit antibody for 2 h at room temperature.

### Quantification of epoxyoctadecenoic acid and dihydroxyoctadec-12-enoic acid

Epoxyoctadecenoic acid (EpOME) and dihydroxyoctadec-12-enoic acid (DHOME) were quantified to investigate the in vivo activity of sEH using a validated high-performance liquid chromatography/mass spectrometry/mass spectrometry method, as described previously [13], [24]. Briefly, a 200-µL plasma sample was spiked with 50 µL of internal standard (200 ng/mL of (±)12,13 DHOME d4), and 2 mL of diethyl ether was added to each sample for liquid-liquid extraction. Detection was achieved using an API-4000 QTRAP (Applied Biosystems, Foster City, CA, USA). Eleven working standards were used at concentrations of 500, 250, 150, 100, 50, 25, 10, 5, 1, 0.5, and 0.1 ng/mL.

### Quantitative real-time polymerase chain reaction

Total RNA was extracted from renal tissues harvested from mice 48 h after the induction of IRI. Cytokine mRNA concentrations were assayed by real-time polymerase chain reaction (PCR). Briefly, total RNA was isolated from kidneys using the RNeasy® kit (Qiagen GmBH, Hilden, Germany) and 1 µg of total RNA was reverse-transcribed using oligo-d(T) primers and AMV-RT Taq polymerase (Promega, Madison, WI, USA). Real-time PCR was performed using assay-on-demand TaqMan® probes and primers for tumor necrosis factor (TNF)- α, monocyte chemoattractant protein (MCP)-1, interleukin (IL)-10, transforming growth factor (TGF)-β1, HIF-1 α, VEGF, erythropoietin (EPO), KDR, c-kit and glyceraldehyde 3-phosphate dehydrogenase (GAPDH) (Applied Biosystems) and an ABI PRISM® 7500 Sequence Detection System (Applied Biosystems). The levels of mRNA expression for each cytokine were normalized with respect to the level of GAPDH mRNA expression.

### Western blot analysis and cytokine assays

The effects of regulating sEH on proteins levels were analyzed by western immunoblotting and cytokine assays. Primary antibodies against sEH, c-kit, and β-actin (Sigma-Aldrich, Saint Louis, MO, USA) were used for western immunoblotting. Briefly, equal amounts (80 µg) of extracted proteins were separated by 10% sodium dodecyl sulfate-polyacrylamide gel electrophoresis and transferred onto Immobilon-FL 0.4 µM polyvinylidene difluoride membranes (Millipore, Bedford, MA). Anti-rabbit IgG (Vector Laboratories, Burlingame, CA) was used as the secondary antibody. Blots were developed using Super Signal West Pico Chemiluminescent Substrate (Pierce, Woburn, MA, USA).

A multiplex cytokine bead array system (Bio-Plex; Bio-Rad) was used to assay for cytokines, according to the manufacturer's instructions.

### Flow cytometry analysis

For quantitative flow cytometry analysis, intrarenal mononuclear cells were isolated from mouse kidney homogenates using Stomacher® 80 Biomaster (Seward Ltd., Worthing, Sussex, UK). Single-cell suspensions were created by passing tissue through a 40-µm cell strainer. Kidneys were resuspended in 36% Percoll (Amersham Pharmacia Biotech, Piscataway, NJ, USA) and overlaid onto 72% Percoll. After centrifuging for 30 min at 1000 *g* at 25°C, renal mononuclear cells were isolated from the interface. Isolated renal mononuclear cells were incubated with directly-conjugated mouse monoclonal antibodies to CD3, CD44, CD45, F4/80, and Gr1 (BD Biosciences). Quantitative fluorescence analysis was performed using a FACSCalibur instrument and CellQuest software (BD Biosciences).

### Statistical analysis

All data are expressed as means±S.E. Group means were compared using Mann-Whitney *U*-tests, one-way ANOVA followed by Tukey's post-hoc analysis, and two-way ANOVA followed by Bonferroni post-hoc analysis using GraphPad Prism® version 4. Statistical significance was determined when the *P* value was <0.05.

## Results

### Regulation of sEH activity reduced IRI in kidneys

**Figure 1:**
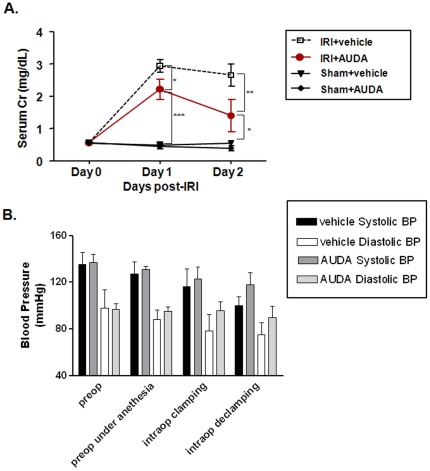
Role of soluble epoxide hydrolase (sEH) activity in ischemia-reperfusion injury (IRI) in kidneys. A: The adamantyl alkyl urea-based sEH inhibitor, 12-(3-adamantan-1-ylureido)-dodecanoic acid (AUDA) reduced IRI in the kidney. All values are given as means±S.E. (*n* = 6 per group for each experiment). Data represent one of three independent experiments. Day 0, before bilateral IRI; day 1, 24 h after bilateral IRI; day 2, 48 h after bilateral IRI (two-way ANOVA with Bonferroni post-testing; *P<0.05; **P<0.01; ***P<0.001). B: Administration of AUDA had no effect on blood pressure during the procedure.

Renal IRI was induced by bilateral clamping of the renal pedicles. Renal function deteriorated substantially after IRI (sham control vs. disease control: day 1 Cr, 0.49±0.06 mg/dL vs. 2.93±0.19 mg/dL, *P*<0.001; day 2 Cr, 0.51±0.02 mg/dL vs. 2.65±0.34 mg/dL, *P*<0.001). To determine the specific role of sEH activity in renal IRI, the specific sEH inhibitor AUDA was administered intraperitoneally (10 mg/kg) 1 h before IRI induction. The severity of renal dysfunction was significantly attenuated in AUDA-treated mice, compared to disease-control mice (day 1 Cr, 2.22±0.31 mg/dL; day 2 Cr, 1.40±0.51 mg/dL, *P*<0.05 compared to C57BL/6 disease-control mice, respectively) (Figure 1A). Blood pressure was monitored during the procedure using a non-invasive blood pressure system. Administration of the sEH inhibitor AUDA had no significant effect on blood pressure compared to administration of vehicle (Figure 1B).

**Figure 2:**
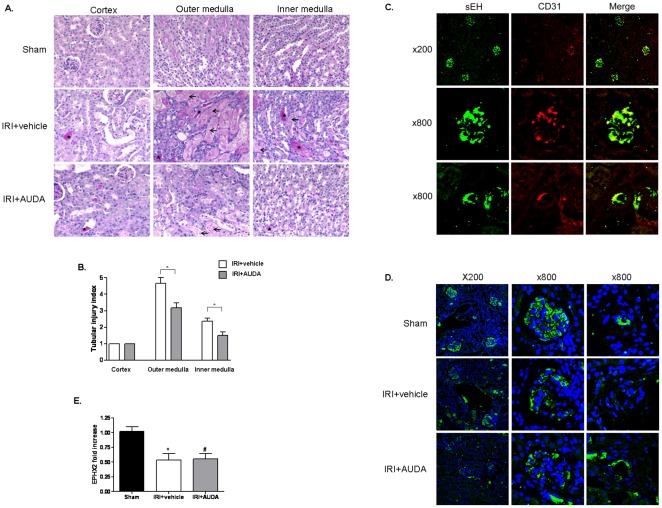
Effects of 12-(3-adamantan-1-ylureido)-dodecanoic acid (AUDA) on renal expression of soluble epoxide hydrolase (sEH) in ischemia-reperfusion injury (IRI) in kidneys. A: Histological changes were consistent with the functional changes (×200). IRI induced tubular necrosis, consisting of disruption and sloughing of tubular epithelial cells. Arrows indicate necrotic tubules, and asterisks indicate tubular casts. Tubular injury was increased in disease-control mice compared to AUDA-treated mice. B: Expression was quantified by a renal pathologist in a blinded fashion (*P<0.05). Scores ranged from 1–5, based on the percentage of tubules affected (1: <10%; 2: 10–25%; 3: 25–50%; 4: 50–75%; 5: >75%). C: sEH was expressed in the endothelium of intraglomerular capillary loops and peritubular capillaries. D and E: Ischemic injury induced the down-regulation of sEH, but AUDA administration had no effect on sEH expression. DAPI was used as counterstaining. *EPHX2,* gene encoding sEH.

Histological examination revealed that the functional changes were paralleled by structural changes. IRI induced tubular necrosis, consisting of the disruption and sloughing of tubular epithelial cells. Tubular necrosis was more prominent in proximal tubules and tubular casts were more obvious in distal tubules (Figure S1). Tubular injury was significantly increased in disease-control mice, but AUDA pretreatment significantly protected against ischemic injury (Figure 2A, 2B). We further confirmed the expression patterns of sEH by immunofluorescence. Confocal microscopy demonstrated prominent expression of sEH in the endothelium of the intraglomerular capillary loops and peritubular capillaries under normal conditions (Figure 2C). Ischemic injury induced down-regulation of sEH, but AUDA administration before IRI had no significant effect on the expression of sEH (Figure 2D, 2E).

### Plasma EpOME and EpOME/DHOME ratio were elevated by sEH inhibition in IRI

**Figure 3:**
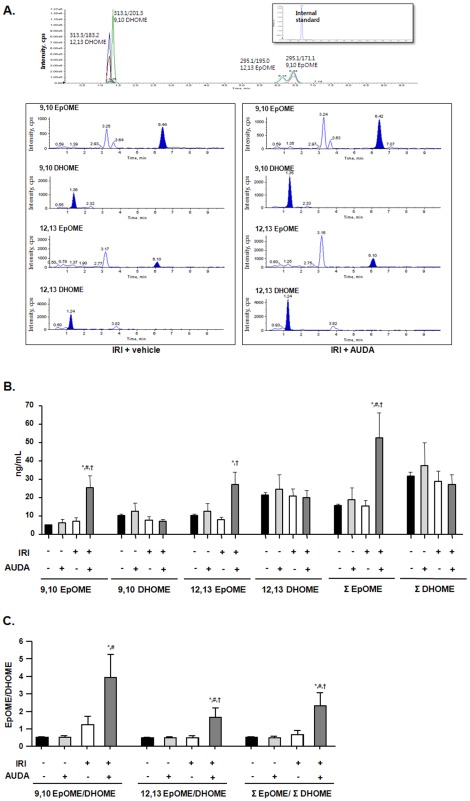
Regulation of soluble epoxide hydrolase (sEH) activity by adamantyl alkyl urea-based sEH inhibitor (AUDA) in renal ischemia-reperfusion injury (IRI). A: Plasma epoxyoctadecenoic acid (EpOME) and dihydroxyoctadec-12-enoic acid (DHOME) levels were quantified to investigate the enzyme activity of sEH. B: 9,10-, 12,13-, and total EpOME plasma concentrations were significantly increased in response to AUDA in renal IRI. C: The EpOME/DHOME ratio was significantly increased (*P<0.05 compared to sham+vehicle; #*P*<0.05 compared to sham+AUDA; †*P*<0.05 compared to IRI+vehicle).

Plasma concentrations of the linoleic acid metabolites EpOME and DHOME were measured to investigate the in vivo activity of sEH (*n* = 6 per group) (Figure 3A). EpOME is a substrate and DHOME is a metabolite of sEH. Ischemic injury had no effect on the plasma concentrations of EpOME and DHOME. However, 9,10-, 12,13- and total EpOME concentrations were significantly increased in response to sEH inhibition by AUDA after IRI (9,10-EpOME [ng/mL]: IRI with vehicle 7.3±2.0 vs. IRI with AUDA 25.4±6.7, *P* = 0.011; 12,13-EpOME [ng/mL]: IRI with vehicle 8.1±1.3 vs. IRI with AUDA 27.2±6.8, *P* = 0.018; total EpOME [ng/mL]: IRI with vehicle 15.5±3.3 vs. IRI with AUDA 52.6±13.5, *P* = 0.013) (Figure 3B). The EpOME/DHOME ratios were also changed by sEH inhibition (9,10-EpOME/DHOME: IRI with vehicle 1.2±0.5 vs. IRI with AUDA 3.9±1.3, *P* = 0.060; 12,13-EpOME/DHOME: IRI with vehicle 0.5±0.1 vs. IRI with AUDA 1.7±0.5, *P* = 0.034; total EpOME/DHOME: IRI with vehicle 0.7±0.2 vs. IRI with AUDA 2.3±0.8, *P* = 0.043) (Figure 3C). However, there were no significant changes in DHOME plasma concentrations.

### Switch from pro- to anti-inflammatory microenvironment in injured kidneys by sEH inhibition

**Figure 4:**
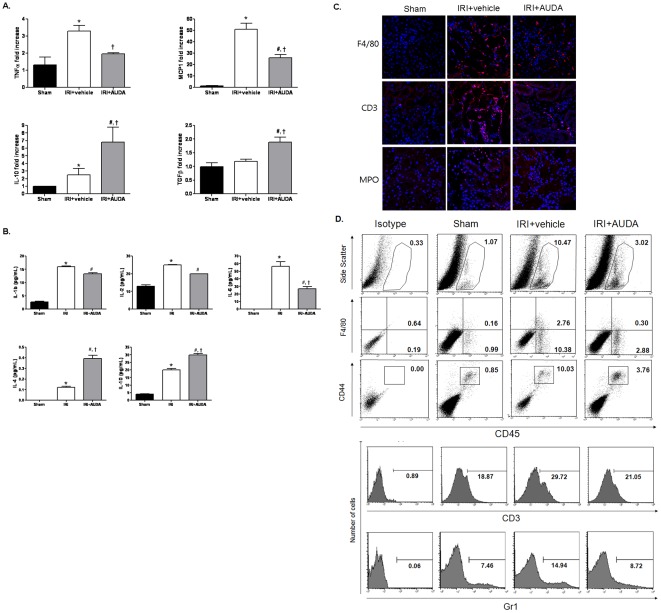
Effects of soluble epoxide hydrolase (sEH) inhibitor on the pro-/anti-inflammatory microenvironment in injured kidneys. A: Proinflammatory cytokines TNF-α and MCP-1 were significantly suppressed, while IL-10 and TGF-β were enhanced by treatment with 12-(3-adamantan-1-ylureido)-dodecanoic acid (AUDA), as shown by real-time PCR. B: The proinflammatory cytokine IL-6 was decreased and the regulatory cytokines IL-4 and IL-10 were augmented by AUDA, as shown by multiplex cytokine assay. (*P<0.05 compared to sham; #*P*<0.05 compared to sham; †*P*<0.05 compared to IRI+vehicle). C: AUDA decreased the infiltration of inflammatory cells (macrophages (F4/80), lymphocytes (CD3), and neutrophils (MPO)) mainly trafficked in the interstitial area. D: AUDA attenuated the infiltration of macrophages/monocytes and T cells expressing CD3, as shown by flow cytometry. F4/80, marker for pan-macrophage; CD44, indicative marker for effector-memory T-cells; CD45, leukocyte common antigen; Gr1, myeloid differentiation antigen.

To evaluate the molecular and cellular mechanisms responsible for the renoprotective effect of the sEH inhibitor, we quantified the cytokines that may influence the extent of renal damage caused by IRI using real-time PCR and a multiplex cytokine bead array system. Real-time PCR analysis revealed up-regulation of proinflammatory cytokines such as TNF-α and MCP-1 associated with the development of IRI in C57BL/6 mice. However, these up-regulated proinflammatory cytokines were significantly suppressed by AUDA treatment. Although IRI also induced up-regulation of the regulatory cytokine IL-10, this change was augmented by AUDA treatment. In addition, AUDA injection significantly enhanced the expression of TGF-β after IRI injury (Figure 4A). Similarly, multiplex cytokine assays revealed that AUDA treatment significantly suppressed the proinflammatory cytokine IL-6, and up-regulated regulatory cytokines such as IL-4 and IL-10 (Figure 4B). Immunofluorescence analysis was performed to identify inflammatory cell infiltrations. Macrophages (F4/80), lymphocytes (CD3), and neutrophils (MPO) mainly trafficked in the interstitial area (Figure 4C), but neutrophil infiltration was relatively low, compared to infiltrations of macrophages or lymphocytes. AUDA reduced the infiltration of inflammatory cells.

Flow cytometric analysis was performed to analyze inflammatory cell trafficking in IRI quantitatively. Macrophages/monocytes expressing F4/80, T cells expressing CD3, and neutrophils expressing Gr1 were all significantly increased in IRI mice compared to sham controls, and these cellular infiltrations were attenuated by AUDA pretreatment (Figure 4D).

### sEH inhibition prevented hypoxic damage via HIF-1α and neovascularization

**Figure 5:**
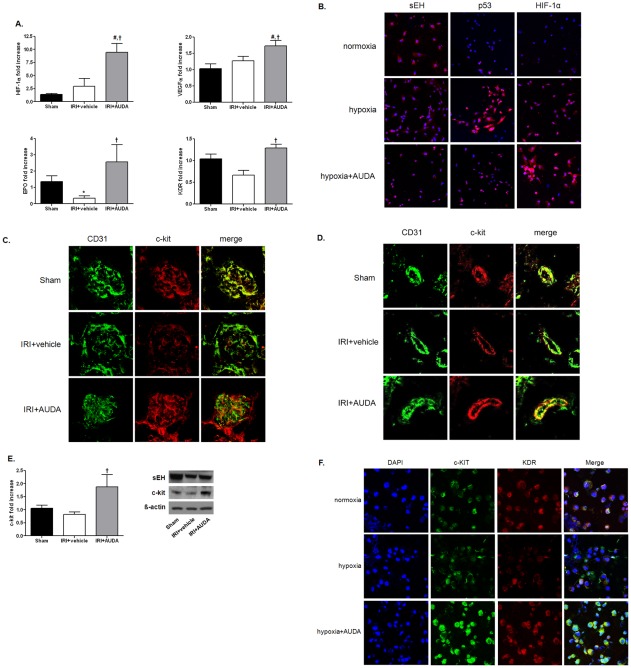
Protective effects of soluble epoxide hydrolase (sEH) inhibitor on hypoxic damage via neovascularization. A: Hypoxia inducible factor (HIF)-1α, vascular endothelial growth factor (VEGF), VEGF receptor-2 (KDR), and erythropoietin (EPO) were enhanced by 12-(3-adamantan-1-ylureido)-dodecanoic acid (AUDA) administration. (*P<0.05 compared to sham; #*P*<0.05 compared to sham; †*P*<0.05 compared to ischemia-reperfusion injury (IRI) +vehicle). B: Hypoxia induced the down-regulation of sEH in human umbilical vein endothelial cells (HUVECs). Cells were incubated with or without AUDA (10 µM) under hypoxic (1% O_2_) or normoxic conditions (20% O_2_) for 24 h. Apoptosis of HUVECs was assessed by p53 expression. Hypoxia induced apoptosis in HUVECs, but AUDA treatment reduced apoptosis associated with enhancement of HIF-1α. DAPI was used for counterstaining (magnification, ×400). C and D: c-kit (CD117) expression decreased after IRI, but was amplified by AUDA administration (magnification, ×800). E: AUDA treatment significantly enhanced c-kit expression. Data represent the results of one of three independent experiments (*n* = 6 per group; †*P*<0.05 compared to IRI+vehicle). F: AUDA treatment increased c-kit and KDR expression levels in HUVECs exposed to hypoxia.

We assessed the expression patterns of molecules associated with endothelial cell migration and neovascularization. HIF-1α was up-regulated by IRI, though the difference was not significant, and it was further enhanced by AUDA administration (Figure 5A). VEGF and EPO, the target genes of HIF-1α, showed similar expression patterns to HIF-1α (Figure 5A). KDR expression was significantly decreased compared to levels in control mice after IRI. AUDA treatment restored the expression levels of these molecules to the levels seen in age-matched injury controls (Figure 5A). When HUVECs were exposed to hypoxia, apoptosis progressed with little change in HIF-1α expression. However, AUDA pretreatment prevented hypoxic HUVECs from undergoing apoptosis, associated with an increase in HIF-1α expression (Figure 5B).

c-kit (CD117) is a cytokine receptor that is expressed on the surface of hematopoietic stem cells. Confocal microscopy revealed that c-kit was also expressed prominently in the endothelium of intraglomerular and peritubular capillaries (Figure 5C, 5D). Levels decreased after IRI, but were significantly amplified by AUDA administration (Figure 5C, 5D, 5E). Enhanced c-kit and KDR expression levels in AUDA-treated hypoxic HUVECs were paralleled by the in vivo IRI model (Figure 5F).

## Discussion

This study evaluated the effects of the adamantyl alkyl urea-based sEH inhibitor AUDA on renal damage caused by IRI. AUDA modulated cytokine secretion and inflammatory cell infiltration at the site of injury, and may have had an effect on renal recovery through activation of HIF-1α and subsequent neovascularization. A few studies have demonstrated a protective effect of sEH inhibitors against IRI such as in stroke [15], [16] or ischemia-induced myocardial damage [17]–[19]. However, this is the first study to demonstrate that sEH inhibition can also effectively protect against IRI-induced renal damage.

IRI is the result of a cascade of reactions, including non-immune injury and immune responses. IRI stimulates the synthesis of pro-inflammatory cytokines, including IL-1, IL-6, and TNF-α [25], [26]. Chemokines also play a role in the pathogenesis of renal injury in post-ischemic kidneys [27]. In contrast, anti-inflammatory cytokines such as IL-10 have been suggested to act as protective or beneficial cytokines, reducing renal injury after IRI [28]. EETs, which are regulated by sEH in endothelial cells, are known to prevent endothelial dysfunction via vasodilatory and non-vasodilatory effects, and anti-inflammatory effects represent an important non-vasodilatory property, distinct from their membrane-hyperpolarizing effect. EETs exert anti-inflammatory effects through the inhibition of nuclear factor-κB-mediated endothelial cell adhesion molecule expression and the prevention of leukocyte adhesion to the vascular wall [10]. In addition, sEH inhibitors indirectly reduced the production of NO, cytokines, and proinflammatory lipid mediators, minimized systemic hypotension, and prevented mortality in a mouse model of septic shock. They also accelerated inflammatory resolution by enhancing lipoxin A4 production [29]. The present study demonstrated that AUDA treatment significantly suppressed the up-regulation of TNF-α and MCP-1 induced by IRI and augmented IL-10 secretion. These findings are consistent with those of previous studies.

Despite the restoration of blood flow during recovery from IRI, an irreversible rarefaction of renal blood vessels persists following the initial resolution of AKI [30]. Furthermore, reductions in vascular density occur following acute IRI, and although the mechanisms mediating vascular loss are unclear, they may relate to the lack of effective vascular repair responses. Previous studies have shown that increased cytochrome P450-derived EETs have proliferative and angiogenic effects. The release of heparin-binding EGF-like growth factor from the endothelial cell surface and the subsequent activation of EGF-receptor-dependent signaling have been suggested to underlie this angiogenesis [31], [32]. HIF-1, which consists of an oxygen-sensitive α-subunit and a constitutively-expressed β-subunit, is one of the key factors in the cellular adaptation to hypoxia [33]. HIF-target genes such as EPO, VEGF, and GLUT-1 are also up-regulated as an adaptive response to hypoxia [34], [35]. VEGF has been suggested to act as a protective or beneficial cytokine, reducing renal injury after IRI [36]. A previous study revealed that increasing EETs reduced cell death via HIF-1α activation [37]. In addition, EETs induce angiogenesis through activation of STAT-3 and VEGF expression [38]. Although HIF-1α expression was only slightly enhanced by IRI or hypoxia in our study, sEH inhibition by AUDA significantly increased HIF-1α expression, with similar patterns of expression seen for HIF-1α target genes. The present study provided the first evidence for a significant increase in the hematopoietic stem cell marker c-kit as a result of sEH inhibition by AUDA.

Several animal studies have suggested that sEH inhibitors may be promising therapeutic agents for cardiovascular disease [15]–[19], [39], [40]. In addition, studies of polymorphisms in the gene encoding sEH (*EPHX2*) have shown that amino-acid substitutions can influence sEH activity in patients with cardiovascular diseases [41]–[44]. As a result, clinical trials have been initiated, including a phase IIa clinical trial of the novel sEH enzyme inhibitor AR9281 (ClinicalTrials.gov Identifier: NCT00847899). In the field of nephrology, sEH inhibitors have also been tested as therapeutic targets in several animal kidney disease models [45]–[47]. We recently confirmed an effect of *EPHX2* genetic polymorphisms on kidney transplantation [14] and IgA nephropathy [13], the most common chronic glomerulonephritis.

Although the results of this study are informative, the study had some limitations. First, there are currently no reports of any adverse or toxic side effects of sEH inhibitors, and no complications or side effects were detected in the current study. In addition, when the study was repeated using half the dose of AUDA (5 mg/kg), a dose-dependent response was obtained (data not shown). However, data on side effects or LD50 values are needed before sEH inhibitors can be used as therapeutic drugs for human diseases. Second, EETs have multiple bioactivities, including direct vasodilation, anti-inflammatory and profibrinolytic effects, and inhibition of vascular smooth muscle proliferation and migration. We examined the vasodilatory effect of AUDA during surgery by measuring blood pressure; however, no difference in blood pressure was detected between the two groups. We only confirmed an anti-inflammatory effect and recovery of endothelial function, but were unable to differentiate between a direct effect and other biologic effects of AUDA.

In conclusion, the results of the current study suggest that sEH inhibitors may have therapeutic potential for the treatment of kidney diseases. However, further studies are needed to clarify the dose-response and adverse-effect profiles of these agents.

## Supporting Information

Figure S1 **Characterization of Injured Tubule.** The brush border of the proximal tubules has an affinity for periodic acid-Schiff (PAS) reagents. Proximal tubules are characterized by abundant cytoplasm and an easily-identifiable brush border, and the amount of cytoplasm, the height of the cells, and the brush border are more prominent in the proximal convoluted portion. The distal tubules and collecting duct cells have less-abundant cytoplasm than the proximal tubular cells, and it is therefore relatively easy to distinguish between proximal and distal tubules by PAS staining. In addition, the collecting duct contains two cell types: principal cells containing aquaporin 2 (AQP 2), with an important function in water reabsorption; and intercalated cells, with high carbonic anhydrase activity 2 (CAII) and an important role in acid-base balance. Immunohistochemical staining of AQP2 (Santa Cruz Biotechnology, Santa Cruz, CA, USA) and CAII (Santa Cruz Biotechnology) confirmed the cells with less-abundant cytoplasm and no brush border as distal tubular cells. Tubular necrosis was more prominent in the proximal tubules, while tubular casts were obvious in the distal tubules in the outer medulla (OM) where the tubular injury was most evident.
(TIF)
