# Supplementary figures and images for: Soluble Epoxide Hydrolase Activity Determines the Severity of Ischemia-Reperfusion Injury in Kidney
Source: PLoS One. 2012 May 10;7(5):e37075. doi: 10.1371/journal.pone.0037075 (PMC3349654; doi:10.1371/journal.pone.0037075)

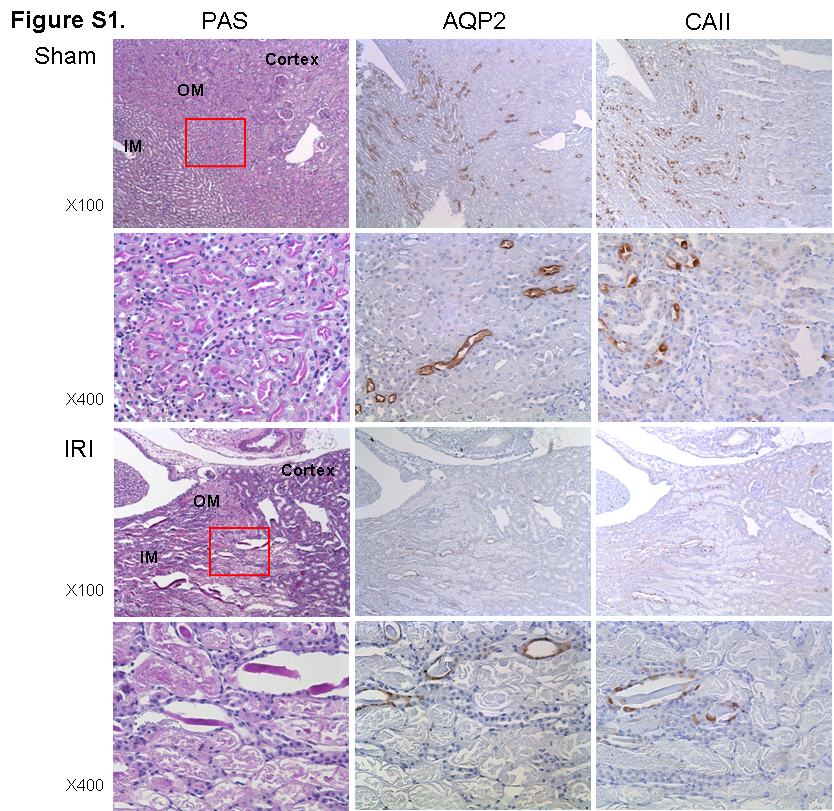

Supplement: Figure S1 — Characterization of Injured Tubule. The brush border of the proximal tubules has an affinity for periodic acid-Schiff (PAS) reagents. Proximal tubules are characterized by abundant cytoplasm and an easily-identifiable brush border, and the amount of cytoplasm, the height of the cells, and the brush border are more prominent in the proximal convoluted portion. The distal tubules and collecting duct cells have less-abundant cytoplasm than the proximal tubular cells, and it is therefore relatively easy to distinguish between proximal and distal tubules by PAS staining. In addition, the collecting duct contains two cell types: principal cells containing aquaporin 2 (AQP 2), with an important function in water reabsorption; and intercalated cells, with high carbonic anhydrase activity 2 (CAII) and an important role in acid-base balance. Immunohistochemical staining of AQP2 (Santa Cruz Biotechnology, Santa Cruz, CA, USA) and CAII (Santa Cruz Biotechnology) confirmed the cells with less-abundant cytoplasm and no brush border as distal tubular cells. Tubular necrosis was more prominent in the proximal tubules, while tubular casts were obvious in the distal tubules in the outer medulla (OM) where the tubular injury was most evident. (TIF) [file pone.0037075.s001.tif]
